# Supplementary material for: HLA-class II restricted TCR targeting human papillomavirus type 18 E7 induces solid tumor remission in mice
Source: Nat Commun. 2024 Mar 13;15:2271. doi: 10.1038/s41467-024-46558-4 (PMC10937927; doi:10.1038/s41467-024-46558-4)

Supplementary Information

HLA-class II restricted TCR targeting HPV18 E7 induces solid tumor remission in mice

Jianting Long[1, #], Xihe Chen[2, #], Mian He[3], Shudan Ou[2], Yunhe Zhao[3], Qingjia Yan[2], Minjun Ma[2], Jingyu Chen[1], Xuping Qin[1], Xiangjun Zhou[2], Junjun Chu[2,*], Yanyan Han[2,*]

[1]Department of Oncology, Cancer Center, the First Affiliated Hospital, Sun Yat-sen University, Guangzhou, P.R. China

[2]HRYZ Biotech Co., Guangzhou, P.R. China

[3]Department of Obstetrics and Gynecology, the First Affiliated Hospital, Sun Yat-sen University, Guangzhou, P.R. China

[#]These authors contributed equally.

[*]Corresponding author. Email: hanyanyan@shhryz.com (Y. H.), chujunjun@shhryz.com (J. C.)

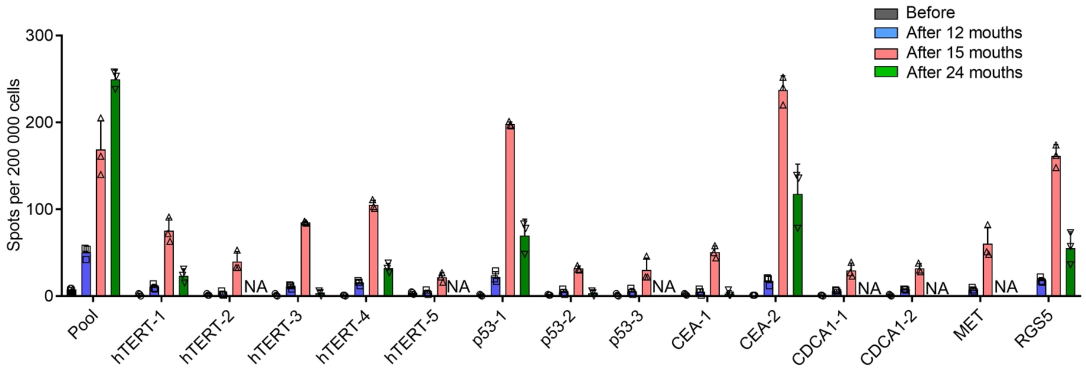

Supplementary Figure 1. **Immune surveillance of patient after MASCT treatments.** *Ex vivo* screening of immune responses against tumor antigens peptides during and after MASCT immunotherapy by IFNγ–ELISPOT assay using T cells from the patient's peripheral blood. Strong and sustained immune responses against telomerase (hTERT), CEA, and HPV18/58 peptides were detected after repeated treatments compared to other peptides. Pool, pooled all antigen peptides. Series numbers indicate different epitopes of the same antigen. NA, not available. Data are shown as the mean ± SD, n=3 technical replicates. Source data are provided as a Source Data file.

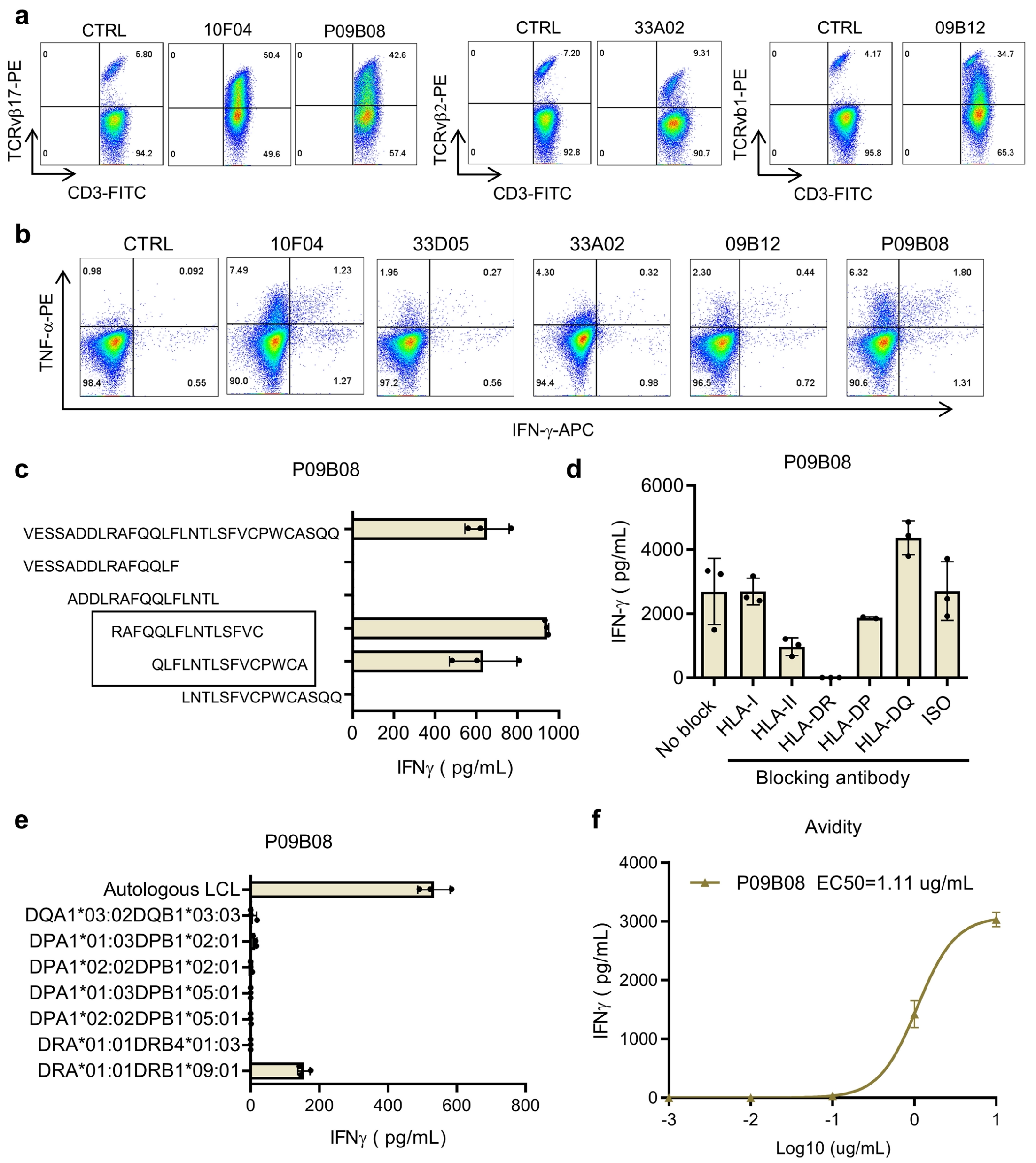

Supplementary Figure 2. Characterization of HPV18 E7 reactive TCRs.

a. Flow cytometry analysis of TCR expression in T cells transduced HPV18 E7 reactive TCRs 10F04, 33A02,09B12, and P09B08, only the 33D05 TCR flow cytometry antibody is not available. **b**. The HPV18 E7 reactive TCRs transduced T cells produced TNFα and IFNγ after *in vitro* stimulation with autologous LCL pulsed with HPV18E7$_{76\text{-}105.}$ **c**. Epitope identification of P09B08. Series truncated peptides derived from HPV18E7$_{76\text{-}105}$ were pulsed on autologous LCLs to stimulate P09B08 transduced T cells. The concentration of IFNγ in the supernatant after overnight co-culture was detected by ELISA. **d**. HLA restriction identification of P09B08. The P09B08 transduced T cells were co-cultured with autologous LCLs pulsed with the HPV18E7$_{76\text{-}105}$ peptide in the presence of blocking antibodies or isotype control. The IFNγ secretion was detected by ELISA and normalized to isotype control. **e**. P09B08 transduced T

cells were co-cultured with HEK-293T cells expressing each HLA-II molecule of the autologous LCL, which were pulsed with HPV18E7$_{76\text{-}105}$ peptide. Autologous LCLs pulsed with HPV18E7$_{76\text{-}105}$ peptide were used as a positive control. IFNγ secretion was detected by ELISA. **f**. Avidity assay of P09B08 TCR transduced T cells. Serial diluted HPV18E7$_{76\text{-}105}$ peptides were used to assess the binding avidity of 10F04 and P09B08. IFNγ secretion was detected by ELISA. Representative results of three biologically independent experiments were shown (**a**, **b**). Data were presented as mean ± SD, n= 3 biologically independent samples for different groups (**c-f**). Source data are provided as a Source Data file.

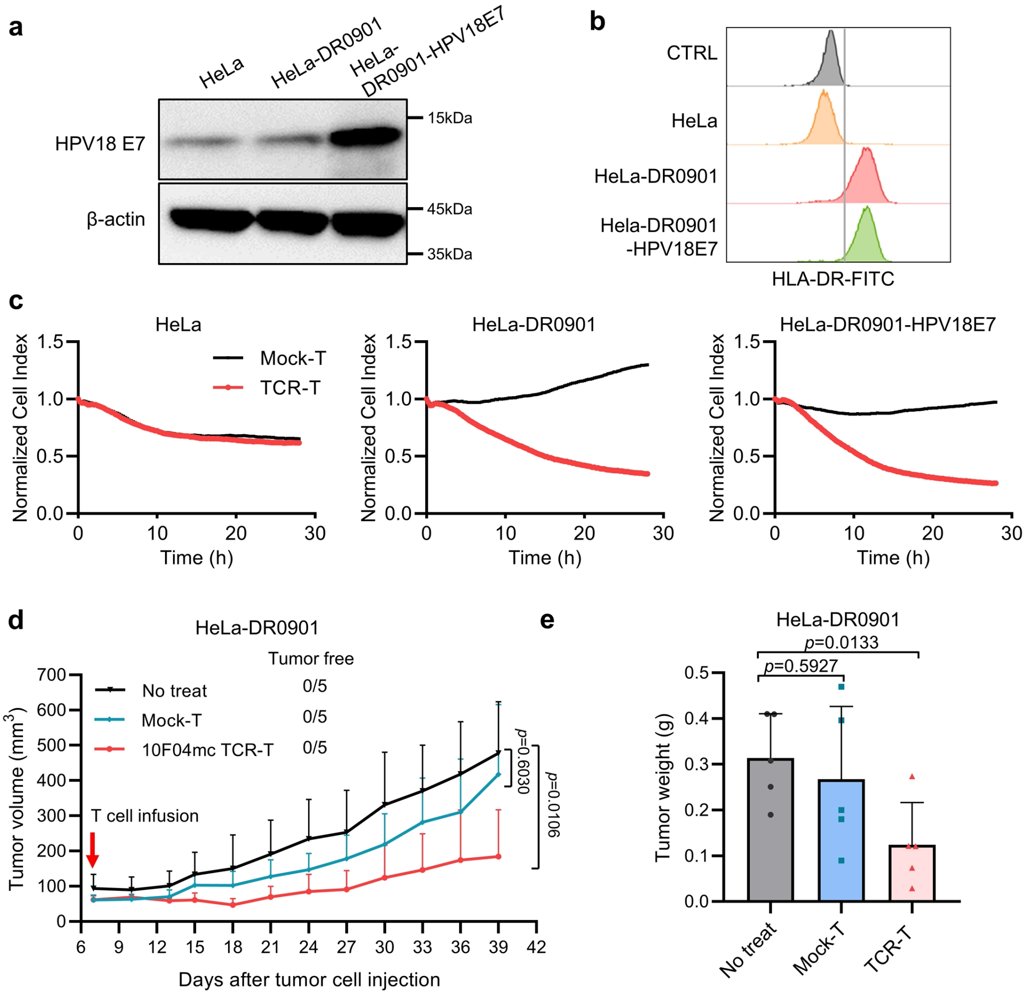

Supplementary Figure 3. Antitumor efficiency of 10F04mc TCR-T cells against tumor cells with different expression levels of HPV18 E7 protein.

a. Western blotting analysis of HPV18E7 in Hela, Hela-DR0901and Hela-DR0901-HPV18E7 cells. **b**. HLA-DR expression analysis of Hela, Hela-DR0901, and Hela-DR0901-HPV18E7 cells was detected by flow cytometry. **c**. *In vitro* killing assay of Hela, Hela-DR0901, and Hela-DR0901-HPV18 cells by 10F04 transduced T cells. **d.** *In vivo* antitumor activity of 10F04mc TCR-T cells in HPV18 E7 endogenous expressed tumor model. NOG mice were injected subcutaneously with $4x10^6$ HeLa-DR0901 cells per mouse on Day 1. TCR-T groups received intravenous injection at a different dose (Low: $1x10^7$, High: $5x10^7$ transduced T cells per mouse, respectively) 6 days after the tumor cells injection. Control group mice received $5x10^7$ Mock-T cells on the same day. The tumor volume was measured by a digital caliper every 2-5 days. Mice were euthanized on Day 48 for tumor isolation and weighing (**e**). Representative western blot images (**a**), flow cytometry data (**b**) and cytotoxicity assay (**c**) of three independent experiments were shown. The *in vivo* animal experiments were repeated twice under similar conditions with similar results, and the representative results were shown. Data were presented as mean + SD, n= 5 mice (**d, e**). Statistical analysis was performed by two-tailed Student's *t* test of the last measurement, with $p < 0.05$ considered significant (**d, e**). Source data are provided as a Source Data file.

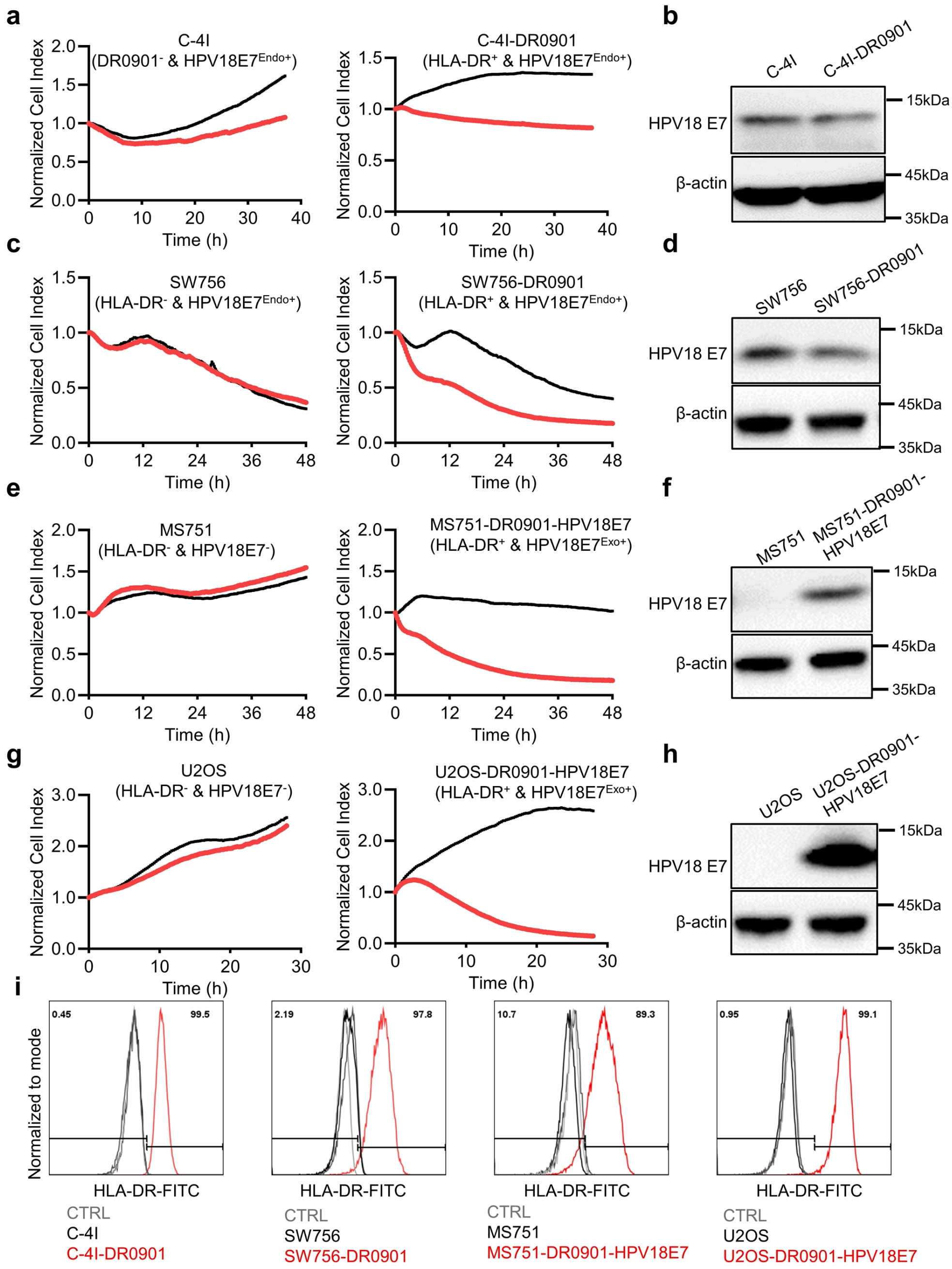

Supplementary Figure 4. Antitumor activity of 10F04mc TCR-T cells in different cell models.

a. *In vitro* cytotoxicity assay of endogenous HPV18-positive human cervical cancer cells c-4I and SW756 (**c**), HLA-DRB1*09:01transduced C-4I and SW756 cells by 10F04 TCR-T cells. HPV18 E7 protein expression was been validated by western blotting (**b**, **d**). **e**. *In vitro* cytotoxicity assay of HPV18-negative human cervical cancer cells MS751 and human osteosarcoma cell line U2OS (**c**), HLA-DRB1*09:01 and HPV18 E7 protein double transduced MS751 and U2OS cells by 10F04 TCR-T cells. HPV18 E7 protein expression was been validated by western blotting (**f**, **h**). **i**. Flow cemetery analysis of HLA-DR expression of those cell lines. Representative killing curve (**a, c, e, g**), western blotting images (**b, d, f, h**) and flow cytometry (**i**) was shown. Source data are provided as a Source Data file.

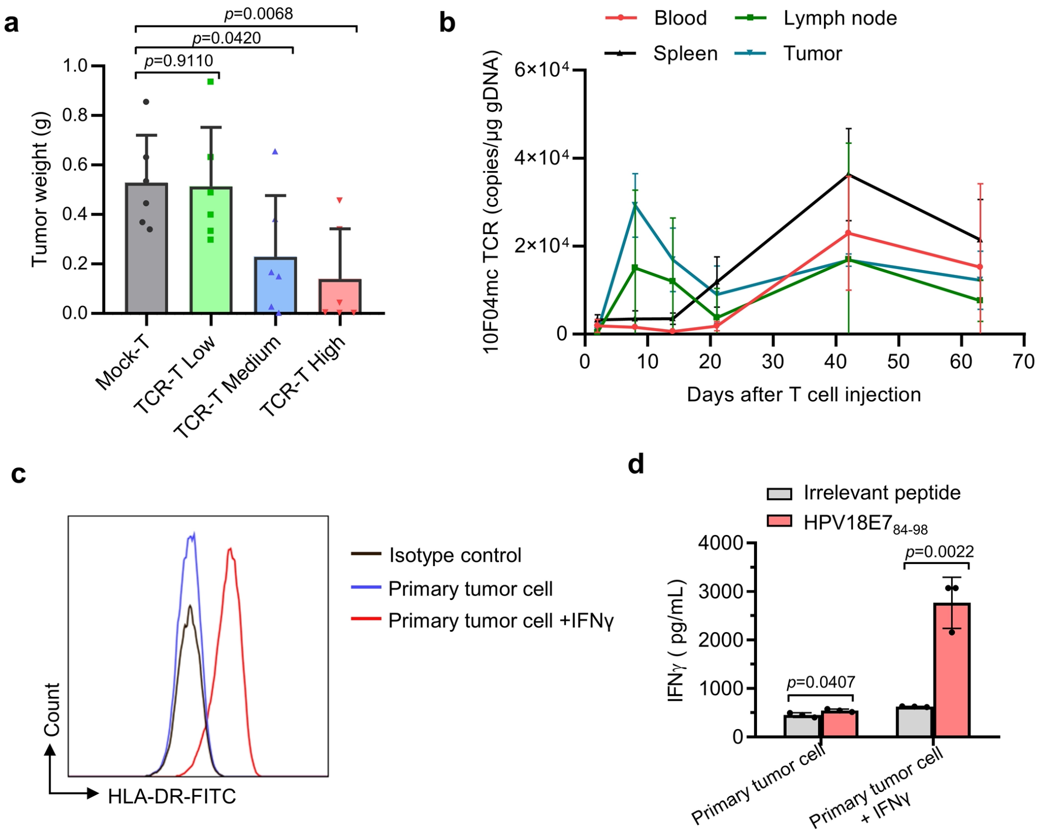

Supplementary Figure 5. Functional analysis of 10F04 TCR transduced human T cells. **a**. Tumors were isolated and weighed after euthanasia at the end of the experiment in **Figure 3e**. N= 6 mice for each group. **b.** *In vivo* distribution and persistence of 10F04mc TCR-T cells. 10F04mc TCR transduced human T cells ($5x10^7$ per mouse) were intravenously injected into HeLa-DR0901-HPV18E7 tumor-bearing NOD mice. Mice were euthanized at each indicated time point and tissues were collected for 10F04mc TCR-T cell quantitative analysis by quantitative PCR. N=6 mice for each time point. **c.** Primary cervical cancer cells were isolated from the surgical specimens of DRA/DRB1*0901$^+$cervical cancer patients. The primary cervical cancer cells were induced by IFNγ for 48 hours to stimulate HLA-II expression. After that, these cells were pulsed with HPV18E7$_{84\text{-}98}$ and co-cultured with 10F04 transduced T cells. HLA-DR expression was detected by flow cytometry (**c**), and IFNγ secretion was detected by ELISA (**d**). N= 3 biologically independent samples for each group (**d**). Data were presented as mean ± SD **(a, b, d)**. Statistical significance was determined by two-tailed Student's *t* test (**a, d**), with $p < 0.05$ considered significant (**d, e**). Source data are provided as a Source Data file.

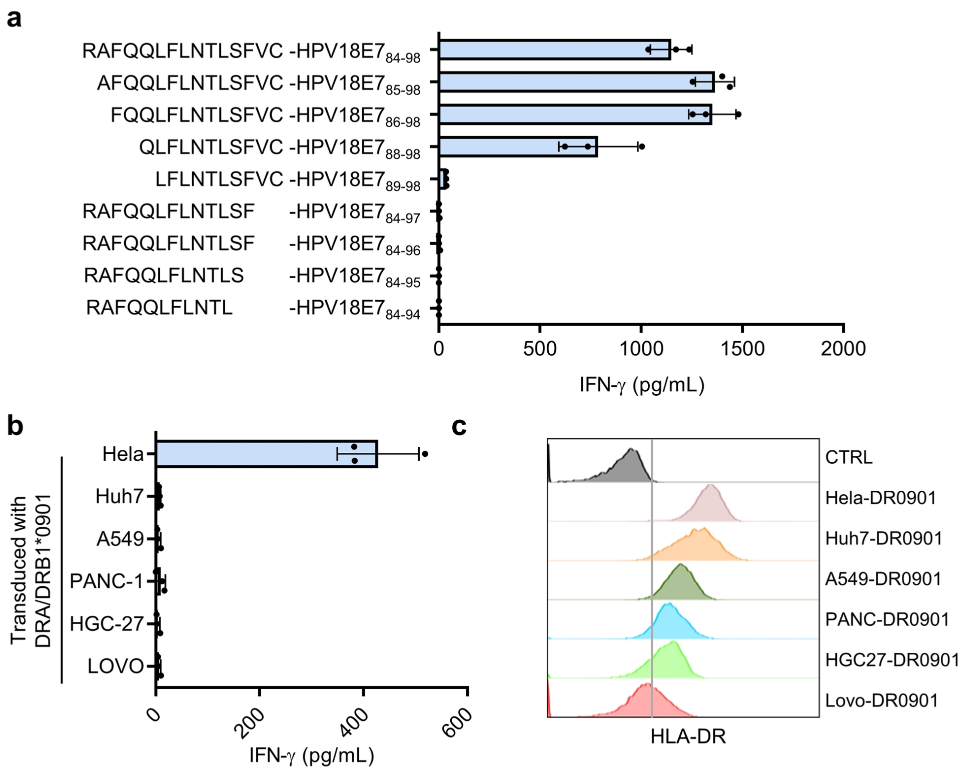

Supplementary Figure 6. Core epitope analysis of 10F04 TCR.

a. The core epitope identification of 10F04 TCR-T cells. Series truncated peptides derived from $HPV18E7_{84-98}$ were pulsed on autologous LCLs to stimulate 10F04 transduced T cells. The IFNγ secretion was detected by ELISA. **b.** Recognition assay of 10F04 TCR-T cells against $HPV18E7^{-}$ but HLA-DRA/DRB1*0901^{+} cancer cell lines. HLA-DRA/DRB1*0901 exogenous overexpressed cancer cells were co-cultured with 10F04 transduced T cells. The IFNγ secretion was detected by ELISA. Endogenously HPV18E7 expressed HeLa cells were set up as positive control. **c**. HLA-DR expression of the cells used in (**b**) was detected by flow cytometry assay. Data were presented as mean ± SD, n=3 biologically independent samples (**a**, **b**). Representative flow cytometry was shown (**c**). Source data are provided as a Source Data file.

a

Core epitope	X	X	F	L	X	T	X	S	F	X	X	Different aa
HPV18-E7	Q	L	F	L	N	T	L	S	F	V	C	-
HPV45-E7	*	*	*	*	S	*	*	*	*	*	*	1/11
HPV70-E7	*	*	*	M	E	*	*	*	*	*	*	2/11
HPV57-E7	*	*	*	*	*	*	*	T	I	*	*	2/11
HPV85-E7	*	*	*	*	G	*	*	*	*	L	*	2/11
HPV59-E7-1	*	*	*	M	D	*	*	*	*	*	*	2/11
HPV59-E7-2	*	*	*	M	D	A	*	*	*	*	*	3/11
HPV221-E7	*	*	L	*	S	Q	*	*	*	*	*	3/11
HPV68-E7-1	*	*	*	M	D	S	*	N	*	*	*	4/11
HPV68-E7-2	L	*	*	M	D	S	*	N	*	*	*	5/11
HPV39-E7-1	*	*	*	M	D	S	*	G	*	*	*	4/11
HPV39-E7-2	K	*	*	M	D	S	*	G	*	*	*	5/11

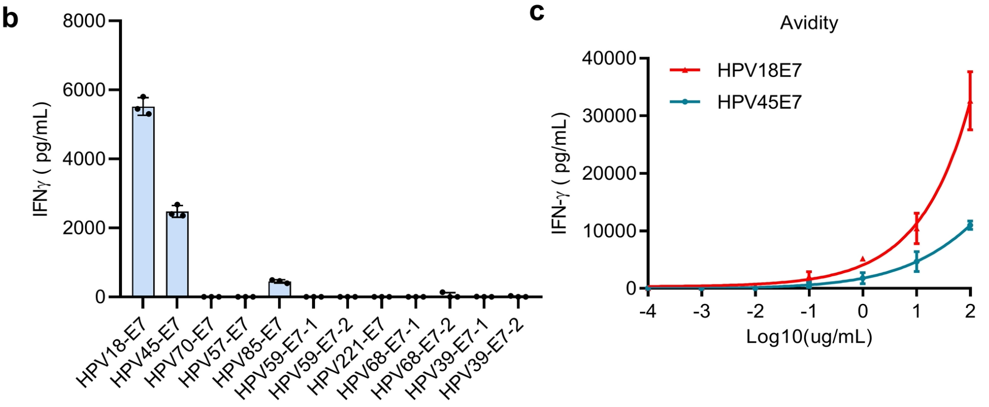

Supplementary Figure 7. Cross-recognition analysis of 10F04mc TCR-T cells against HPV-derived epitopes.

a. HPV family sequence homology between the E7 proteins in the Core epitope of 10F04mc TCR. Amino acids on an orange background indicate the core motif. **b**. 10F04mc TCR cross-reactive to HPV47E7. 10F04mc transduced T cells were stimulated by LCLs pulsed with HPV18E7$_{88\text{-}98}$, HPV45$_{89\text{-}99}$, HPV70$_{91\text{-}101}$, HPV57$_{78\text{-}88}$, HPV85$_{91\text{-}101}$, HPV59$_{90\text{-}100}$, HPV221$_{72\text{-}82}$, HPV68$_{92\text{-}102}$, and HPV39$_{91\text{-}101}$. The concentration of IFNγ in the supernatant after overnight co-culture was detected by ELISA. **c**. The binding avidity of 10F04mc TCR to HPV18E7$_{88\text{-}98}$ and HPV45$_{89\text{-}99}$. IFNγ secretion was detected by ELISA. Representative results were shown and presented as mean ± SD, n=3 biologically independent samples (**b**), n=2 biologically independent samples (**c**). Source data are provided as a Source Data file.

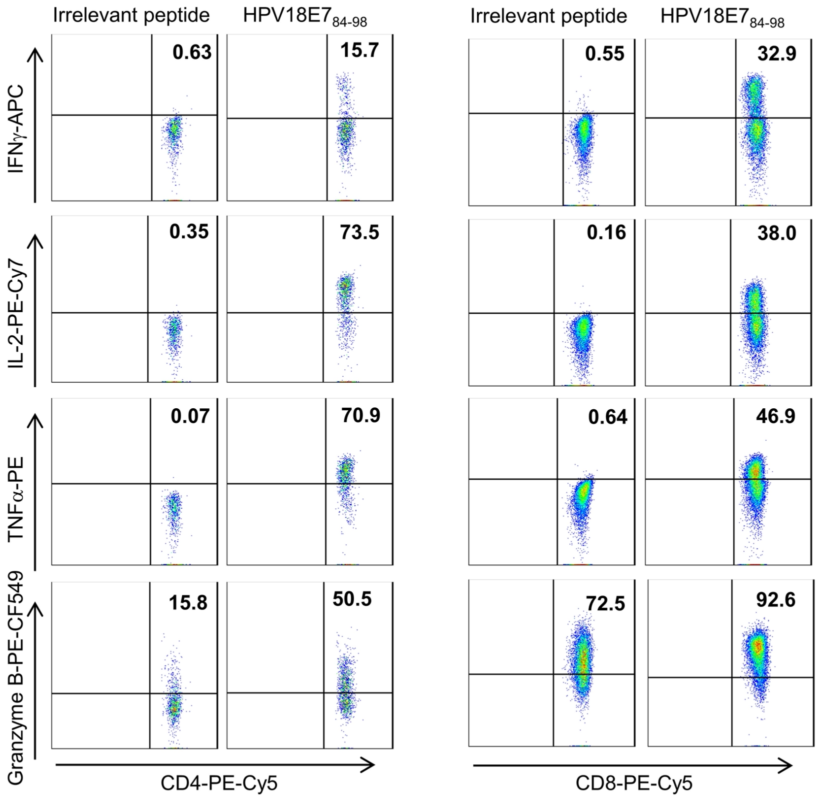

Supplementary Figure 8. Multiple cytokines expression of 10F04mc TCR transduced T cells after stimulation with HPV18E7$_{76\text{-}105}$ peptide.

The 10F04mc transduced T cells produced IFNγ, IL-2, TNFα, and Granzyme B after *in vitro* stimulation.10F04mc transduced T cells were co-cultured with K562-DRA/DRB1*09:01 pulsed with HPV18E7$_{84\text{-}98}$ or irrelevant peptide. Intracellular IFNγ, IL-2, TNFα, and Granzyme B production were analyzed respectively in the CD4^{+} T cells and CD8^{+} T cells. Representative flow cytometry was shown.

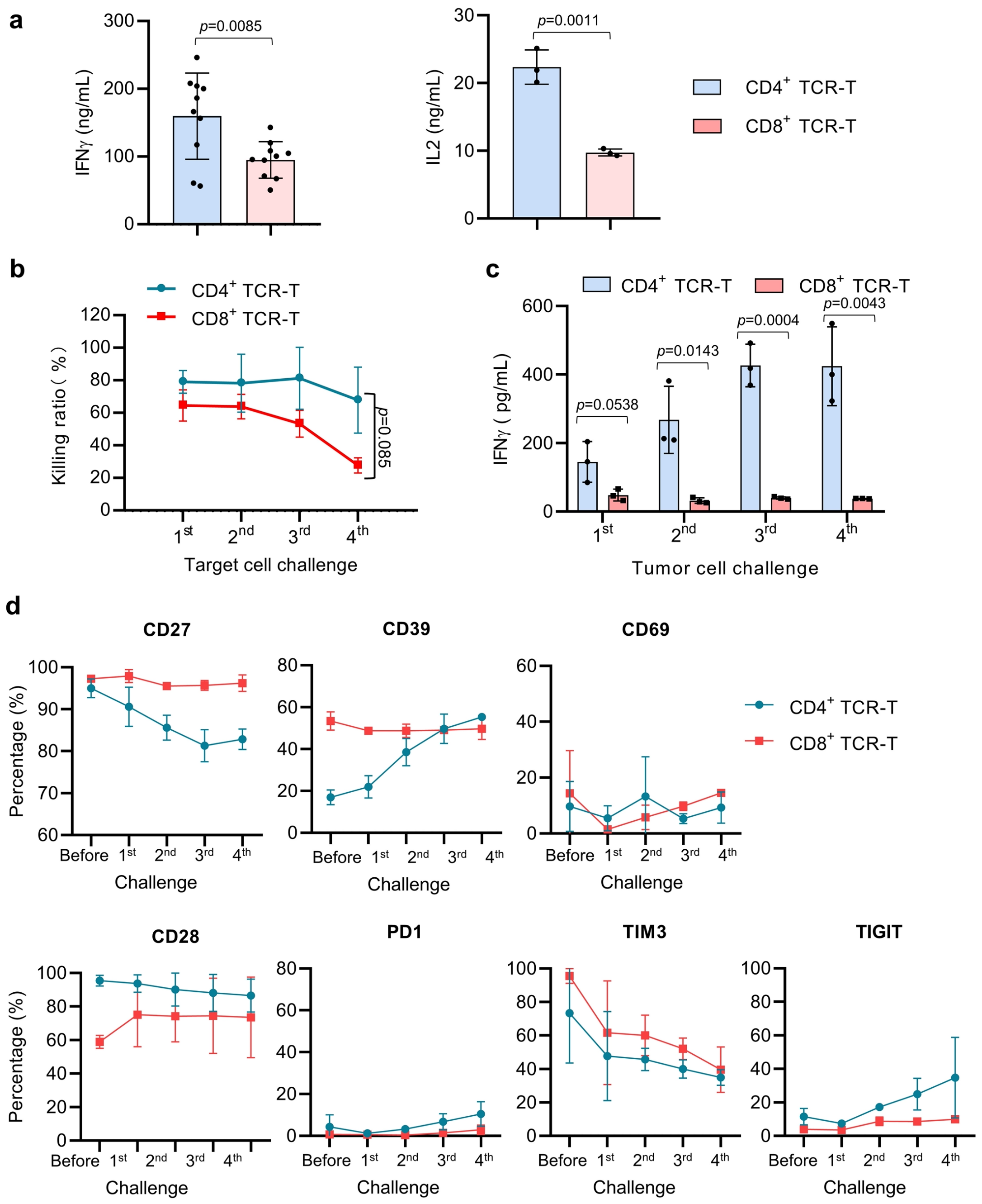

Supplementary Figure 9. Characterization of CD4 and CD8 10F04mc TCR-T cells after repeat challenges with tumor cells.

a. IFNγ and IL-2 secretion of 10F04mc transduced $CD4^+$T and $CD8^+$ T cells. $CD4^+$ T cells and $CD8^+$ T cells were separated by positive selection after 10F04m transduction, and then co-cultured with K562-DRA/DRB1*09:01 pulsed with HPV18E7$_{76\text{-}105}$ peptide. The concentration of IFNγ and IL-2 in the supernatant was detected by ELISA. N= 10 biologically independent samples for INFγ assay and n= 3 biologically independent samples for IL-2 assay. **b**. Summarization of the killing ratio of 4 independent repeat challenge experiments in **Figure 5c**. N= 4 for each group. **c**. 10F04mc transduced $CD4^+$ TCR-T cells produced more IFNγ than CD8+TCR-T cells during the repeat challenge experiment. IFNγ secretion was detected by ELISA. N= 4 biologically independent samples for each group. **d**. The expression of the cell

surface markers CD27, CD39, CD69, CD28, PD1, TIM3and TIGIT on 10F04mc transduced $CD4^+$ TCR-T cells and $CD8^+$ TCR-T cells in repeat challenge experiment. N= 2 biologically independent samples. Data were shown as means ± SD (**a**-**d**). Statistical significance was determined by two-tailed Student's *t* test (**a**-**c**), with $p < 0.05$ considered significant. Source data are provided as a Source Data file.

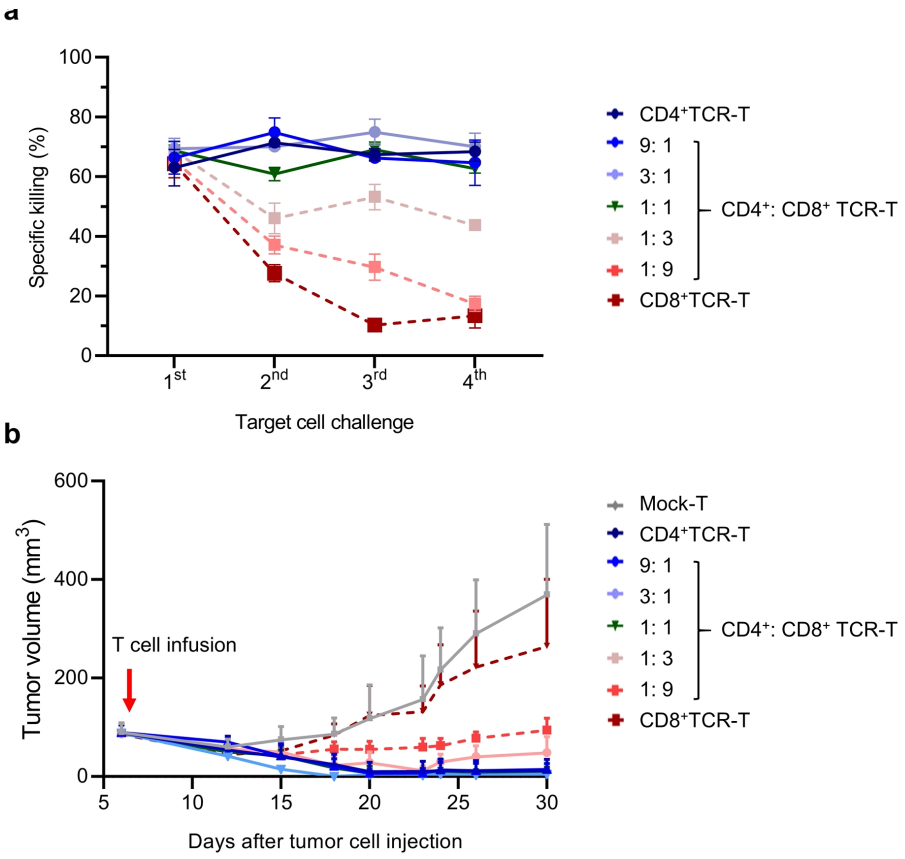

Supplementary Figure 10. Characterization of 10F04mc TCR transduced $CD4^+$ and $CD8^+$ T cell function *in vitro* and *in vivo*.

a. *In vitro* killing assay for a series mixture of $CD4^+$ and $CD8^+$ 10F04mc TCR-T cells. The 10F04mc transduced $CD4^+$ and $CD8^+$ TCR-T cells were premixed at indicated ratios and repeat challenged with HeLa-DR0901 cells. The target cell-killing ability was evaluated by the RTCA system. **b.** *In vivo* antitumor activity assay for a series mixture of $CD4^+$ TCR-T and $CD8^+$ TCR-T cells. The 10F04mc transduced $CD4^+$ and $CD8^+$ TCR-T cells were premixed at indicated ratios and a total number of $5x10^7$ TCR-T cells were administrated to HeLa-DR0901-HPV18E7 tumor-bearing NOG mice 6 days after tumor cell injection. The tumor volume was measured every 3-5 days. Data presented as mean ± SD, n= 3 biologically independent samples (**a**); or mean + SD (**b**), n=4 mice for each group.

Supplementary Table 1. HLA typing information of the LCLs in Figure 4c.

LCL lines	Class I						Class II									
	HLA-A		HLA-B		HLA-C		HLA-DRB1		HLA-DQA1		HLA-DQB1		HLA-DPA1		HLA-DPB1	
LCL-3	A*02:03	A*11:02	B*27:04	B*38:02	C*07:02	C*12:02	DRB1*09:01	DRB1*13:12	DQA1*01:04	DQA1*05:03	DQB1*03:01	DQB1*05:03	DPA1*02:02	DPA1*04:01	DPB1*05:01	DPB1*13:01
LCL-19	A*11:01	A*26:01	B*13:01	B*46:01	C*01:02	C*03:04	DRB1*14:54	DRB1*09:01	DQA1*01:04	DQA1*03:02	DQB1*03:03	DQB1*05:02	DPA1*02:02	DPA1*04:01	DPB1*05:01	DPB1*13:01
LCL-25	A*02:07	A*30:01	B*13:02	B*46:01	C*01:02	C*06:02	DRB1*07:01	DRB1*09:01	DQA1*02:01	DQA1*03:02	DQB1*02:02	DQB1*03:03	DPA1*01:03	DPA1*02:02	DPB1*04:01	DPB1*05:01
LCL-47	A*24:02	A*26:01	B*35:01	B*51:01	C*03:03	C*14:02	DRB1*09:01	DRB1*12:02	DQA1*03:02	DQA1*06:01	DQB1*03:01	DQB1*03:03	DPA1*02:02	DPA1*04:01	DPB1*13:01	DPB1*135:01
LCL-10	A*02:06	A*11:01	B*15:02	B*35:01	C*07:02	C*08:01	DRB1*12:02	DRB1*15:01	DQA1*01:02	DQA1*06:01	DQB1*03:01	DQB1*06:02	DPA1*01:03	DPA1*02:02	DPB1*02:01	DPB1*21:01
LCL-14	A*02:01	A*24:02	B*13:01	B*54:01	C*03:04	C*14:02	DRB1*04:05	DRB1*12:02	DQA1*03:03	DQA1*06:01	DQB1*03:01	DQB1*04:01	DPA1*02:02	DPA1*02:02	DPB1*05:01	DPB1*05:01
LCL-15	A*11:01	A*24:02	B*13:01	B*40:01	C*03:04	C*04:01	DRB1*15:01	DRB1*12:01	DQA1*01:02	DQA1*05:05	DQB1*03:01	DQB1*06:01	DPA1*01:03	DPA1*02:02	DPB1*02:01	DPB1*03:01
LCL-17	A*11:01	A*24:02	B*13:01	B*54:01	C*01:02	C*03:04	DRB1*04:05	DRB1*15:01	DQA1*01:02	DQA1*03:03	DQB1*04:01	DQB1*06:01	DPA1*01:03	DPB1*01:03	DPB1*02:01	DPB1*03:01
LCL-22	A*11:01	A*11:01	B*13:01	B*51:02	C*03:04	C*14:02	DRB1*12:02	DRB1*15:01	DQA1*01:02	DQA1*01:02	DQB1*05:02	DQB1*06:01	DPA1*01:03	DPA1*02:02	DPB1*03:01	DPB1*05:01
LCL-23	A*02:07	A*11:01	B*13:01	B*15:02	C*03:04	C*08:01	DRB1*15:01	DRB1*15:01	DQA1*01:02	DQA1*01:02	DQB1*06:01	DQB1*06:01	DPA1*01:03	DPA1*02:02	DPB1*02:01	DPB1*05:01
LCL-24	A*24:02	A*24:02	B*40:02	B*40:02	C*03:03	C*03:04	DRB1*11:01	DRB1*12:02	DQA1*05:05	DQA1*06:01	DQB1*03:01	DQB1*03:01	DPA1*01:03	DPA1*02:02	DPB1*05:01	DPB1*48:01
LCL-29	A*02:03	A*02:06	B*38:02	B*51:01	C*07:02	C*14:02	DRB1*12:02	DRB1*14:04	DQA1*01:04	DQA1*06:01	DQB1*03:01	DQB1*05:03	DPA1*02:02	DPA1*04:01	DPB1*05:01	DPB1*107:01
LCL-31	A*11:01	A*33:03	B*38:02	B*58:01	C*03:02	C*07:02	DRB1*14:18	DRB1*15:02	DQA1*01:02	DQA1*01:04	DQB1*05:02	DQB1*05:03	DPA1*02:02	DPA1*02:02	DPB1*02:02	DPB1*05:01
LCL-34	A*11:01	A*33:03	B*15:01	B*58:01	C*03:02	C*04:01	DRB1*03:01	DRB1*15:01	DQA1*01:02	DQA1*05:01	DQB1*02:01	DQB1*06:01	DPA1*02:02	DPA1*02:02	DPB1*05:01	DPB1*05:01
LCL-48	A*02:01	A*30:01	B*13:02	B*15:11	C*03:03	C*06:02	DRB1*07:01	DRB1*14:54	DQA1*01:04	DQA1*02:01	DQB1*02:02	DQB1*05:03	DPA1*02:02	DPA1*02:02	DPB1*02:01	DPB1*05:01
LCL-54	A*11:01	A*11:01	B*39:05	B*55:02	C*01:02	C*07:02	DRB1*08:03	DRB1*14:54	DQA1*01:03	DQA1*01:04	DQB1*05:03	DQB1*06:01	DPA1*02:01	DPA1*02:02	DPB1*05:01	DPB1*13:01

Uncropped scans of all western blots

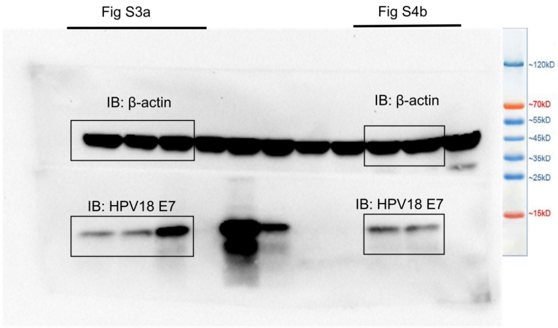

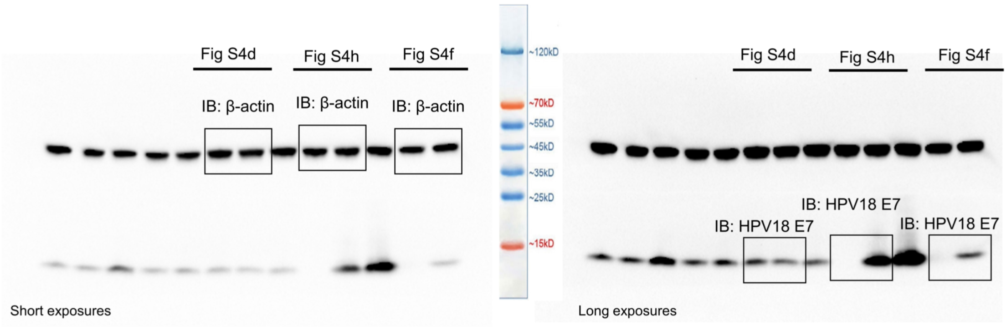

Supplement: Supplementary file 1 — Supplementary Information [file 41467_2024_46558_MOESM1_ESM.pdf]
