# Supplementary material for: HLA-class II restricted TCR targeting human papillomavirus type 18 E7 induces solid tumor remission in mice
Source: Nat Commun. 2024 Mar 13;15:2271. doi: 10.1038/s41467-024-46558-4 (PMC10937927; doi:10.1038/s41467-024-46558-4)
Supplement: Supplementary file 3 — Description of Additional Supplementary Files [file 41467_2024_46558_MOESM3_ESM.pdf]

Supplementary Movie 1. Real-time *in vitro* cytotoxicity assay of non-transduced control human T cells.

HeLa-DR0901 cells were plated onto the Real-Time Cell Analyzer (RTCA) plate wells for adhesion before co-culturing with Mock-T cells. The RTCA was used to capture real-time in vitro cytotoxicity time-lapse videos, each spanning 14 hours with frames taken at 30-minute intervals.

Supplementary Movie 2. Real-time *in vitro* cytotoxicity assay of 10F04mc TCR transduced human T cells.

HeLa-DR0901 cells were plated onto the Real-Time Cell Analyzer (RTCA) plate wells for adhesion before co-culturing with 10F04mc TCR-T cells. The RTCA was used to capture real-time in vitro cytotoxicity time-lapse videos, each spanning 14 hours with frames taken at 30-minute intervals.
