# Supplementary material for: HLA-class II restricted TCR targeting human papillomavirus type 18 E7 induces solid tumor remission in mice
Source: Nat Commun. 2024 Mar 13;15:2271. doi: 10.1038/s41467-024-46558-4 (PMC10937927; doi:10.1038/s41467-024-46558-4)
Supplement: Supplementary file 6 — Reporting Summary [file 41467_2024_46558_MOESM6_ESM.pdf]

nature portfolio

Corresponding author(s): Yanyan Han, Junjun Chu

Last updated by author(s): Feb 8, 2024

Reporting Summary

Nature Portfolio wishes to improve the reproducibility of the work that we publish. This form provides structure for consistency and transparency in reporting. For further information on Nature Portfolio policies, see our Editorial Policies and the Editorial Policy Checklist.

Statistics

For all statistical analyses, confirm that the following items are present in the figure legend, table legend, main text, or Methods section.

n/a	Confirmed	
☐	☒	The exact sample size (*n*) for each experimental group/condition, given as a discrete number and unit of measurement
☐	☒	A statement on whether measurements were taken from distinct samples or whether the same sample was measured repeatedly
☐	☒	The statistical test(s) used AND whether they are one- or two-sided<br>*Only common tests should be described solely by name; describe more complex techniques in the Methods section.*
☒	☐	A description of all covariates tested
☐	☒	A description of any assumptions or corrections, such as tests of normality and adjustment for multiple comparisons
☐	☒	A full description of the statistical parameters including central tendency (e.g. means) or other basic estimates (e.g. regression coefficient) AND variation (e.g. standard deviation) or associated estimates of uncertainty (e.g. confidence intervals)
☐	☒	For null hypothesis testing, the test statistic (e.g. *F*, *t*, *r*) with confidence intervals, effect sizes, degrees of freedom and *P* value noted<br>*Give P values as exact values whenever suitable.*
☒	☐	For Bayesian analysis, information on the choice of priors and Markov chain Monte Carlo settings
☒	☐	For hierarchical and complex designs, identification of the appropriate level for tests and full reporting of outcomes
☒	☐	Estimates of effect sizes (e.g. Cohen's *d*, Pearson's *r*), indicating how they were calculated

Our web collection on statistics for biologists contains articles on many of the points above.

Software and code

Policy information about availability of computer code

Data collection	The number of spot-forming units of ELISPOT assay was detected by C.T.L. Immuno Spot S6 Analyzer. Cytotoxicity assays data were collected on an RTCA analyzer by xCELLigence software (ACEA Biosciences). FACS data was obtained on BD FACS Canto II flow cytometer or Attune NxT flowcytometer (ThermoFIsher Scientific)
Data analysis	ELISPOT assay data were analyzed by Immuno Spot v6.0 software. Cytotoxicity assays data were analyzed by xCELLigence software (ACEA Biosciences). FACS data were analyzed with Flowjo software v10.8.1. All statistical analyses were performed using GraphPad Prism version 8.0.1 (GraphPad Software)

For manuscripts utilizing custom algorithms or software that are central to the research but not yet described in published literature, software must be made available to editors and reviewers. We strongly encourage code deposition in a community repository (e.g. GitHub). See the Nature Portfolio guidelines for submitting code & software for further information.

Data

Policy information about availability of data

All manuscripts must include a data availability statement. This statement should provide the following information, where applicable:

- Accession codes, unique identifiers, or web links for publicly available datasets
- A description of any restrictions on data availability
- For clinical datasets or third party data, please ensure that the statement adheres to our policy

Provide your data availability statement here.

Research involving human participants, their data, or biological material

Policy information about studies with human participants or human data. See also policy information about sex, gender (identity/presentation), and sexual orientation and race, ethnicity and racism.

Reporting on sex and gender	Cervical cancer patients derived tumor tissue sample and PBMCs were collected from female donors. 10F04 TCR was cloned from a 47-year old female donor who has been diagnosed with HPV positive metastatic cervical squamous cell carcinoma in 2011 .
Reporting on race, ethnicity, or other socially relevant groupings	Human tissue or PBMC samples were derived from Asians.
Population characteristics	The PBMCs used for TCR transduction in this study were obtained from both male and female healthy donors aged 18 to 25 years.
Recruitment	Operable cervical squamous cell carcinoma, adenocarcinoma, adenosquamous carcinoma, and other cervical subtypes patients' surgical specimens specimens and PBMCs were collected from the First Affiliated Hospital, Sun Yat-sen University.
Ethics oversight	Human tissue samples and blood were collected following approval by the Ethics Committee (2014-01) of the First Affiliated Hospital, Sun Yat-sen University.

Note that full information on the approval of the study protocol must also be provided in the manuscript.

Field-specific reporting

Please select the one below that is the best fit for your research. If you are not sure, read the appropriate sections before making your selection.

☒ Life sciences ☐ Behavioural & social sciences ☐ Ecological, evolutionary & environmental sciences

For a reference copy of the document with all sections, see nature.com/documents/nr-reporting-summary-flat.pdf

Life sciences study design

All studies must disclose on these points even when the disclosure is negative.

Sample size	No statistical methods were used to pre-determine the sample size. Instead, sample size was chosen based on the previous experience to obtain statistical significance and reproducibility
Data exclusions	We did not exclude any data in this paper.
Replication	Most experiments were repeated at least three times independently as indicated in the figure legends. All animal experiments were repeated at least for two times. All attempts at replication were successful. Some experiments using patients islets were performed once because of the shortage of islets.
Randomization	All animal experiments were randomized. Both males, females, and age-matched mice were used. For cell culture experiments, cells were prepared and randomly distributed into each well of the plates for different experiments.
Blinding	Investigators were blinded to group allocation during animal experiment data collection. For other cell culture experiments experiments, they were not blinded due to different treatments or experiments performed by the same person.

Reporting for specific materials, systems and methods

We require information from authors about some types of materials, experimental systems and methods used in many studies. Here, indicate whether each material, system or method listed is relevant to your study. If you are not sure if a list item applies to your research, read the appropriate section before selecting a response.

Materials & experimental systems

n/a	Involved in the study
☐	☒ Antibodies
☐	☒ Eukaryotic cell lines
☒	☐ Palaeontology and archaeology
☐	☒ Animals and other organisms
☒	☐ Clinical data
☒	☐ Dual use research of concern
☒	☐ Plants

Methods

n/a	Involved in the study
☒	☐ ChIP-seq
☐	☒ Flow cytometry
☒	☐ MRI-based neuroimaging

Antibodies

Antibodies used

anti-CD3 (OKT3, Biolegend, cat. 317315), anti-CD28 (CD28.2, Biolegend, cat. 302914). Hamster anti-mouse TCRchain antibody (H57-597, BD Biosciences, cat. 553171) or anti-human TCR Vβ17 Antibody (E17.5F3.15.13, BECKMAN, cat. IM2048). anti-human CD3-APC-Cy7 (SK7, BD Biosciences, cat. 557832), anti-human CD8-PercP (SK1, Biolegend, cat. 344708), anti-human CD8-PE-Cy5 (Hit8a, Biolegend, cat. 300910), anti-human CD4-PE-Cy7 (A161A1, Biolegend, cat. 357410), anti-human CD4-PE-Cy5 (RPA-T4, Biolegend, cat. 555348), CD3-Alexa Flour700 (SK7, Biolegend, cat. 344822), anti-mouse TCRchain-PE(H57-597, BD Biosciences, cat. 553172), anti-mouse TCRchain-FITC (H57-597, BD Biosciences, cat. 553171) and anti-human TCR Vβ17-PE (E17.5F3.15.13, BECKMAN cat. IM2048), anti-human TCR Vβ2-PE (MPB2D5, BECKMAN, cat. IM2213), anti-human TCR Vβ1-PE (REA662, Miltenyi, cat. 130-110-018), anti-human HLA-DR-FITC (G46-6, BD, cat. 555811), anti-human CD27-BV421 (M-T271, Biolegend, cat. 356418), anti-human CD39-BV510 (A1, BD, cat. 567526), , anti-human PD1-BV711 (MIH4, BD, cat.740814) , anti-human CD3-BV570 (UCHT1, Biolegend, cat. 300436), anti-human CD4-ef450 (SK3, invitrogen, cat. 48-0047-42), anti-human CD4-V500 (RPA-T4, BD, cat.560768), anti-human CD8-AF700 (RPA-T8, BD, cat. 557945), anti-human CD8-APC-Cy7(RPA-T8, BD, cat. 557834), anti-mouse TCRvβ chain-APC-Cy7 (H57-597, BD, cat.560656). anti-human IFNγ-APC (B27, BD Biosciences, cat. 554702), anti-human TNFα-PE (MAb11, BD Biosciences, cat. 559321), anti-human Granzyme B-FITC (GB11, BD Biosciences, cat. 560211), anti-human Granzyme B-PE-CF594 (GB11, BD Biosciences, cat. 562062), anti-human IL-2-PE-Cy7 (MQ1-17H12, BD Biosciences, cat. 560707). anti-HPV18 E7 antibody (8E2, abcam, ab100953, dilution: 1:1000), anti-β-actin antibody (2D4H5, proteintech, 66009-1-Ig, dilution: 1:5000), HRP-conjugated Affinipure Goat Anti-Mouse IgG(H+L) (proteintech, SA00001-1, dilution: 1:2000). anti-Human HLA-A, B, C (W6/32, Biolegend, 311423, final Co. 50 μg/ml), anti-Human HLA-DR, DP, DQ (Tu39, BD Biosciences, cat. 555556, final Co. 50 μg/ml), anti-HLA-DR (G46-6, BD Biosciences, cat. 555809, final Co. 50 μg/ml), anti-HLA-DP (B7/21, Abcam, cat. ab20897, final Co. 50 μg/ml) and anti-HLA-DQ (SPL-L3, Abcam, cat. Ab23632, final Co. 50 μg/ml).Neutralizing CD4 Monoclonal Antibody (RPA-T4, eBioscience, Cat. 16-0049-85, final Co. 5 μg/ml)

Validation

All antibodies used in this study are from commercial sources. All commercial antibodies have been tested for specificity by their respective suppliers. Antibody validation and validation criteria are available on suppliers' websites.

Eukaryotic cell lines

Policy information about cell lines and Sex and Gender in Research

Cell line source(s)

B95.8, HEK-293T, and HeLa cell lines were purchased from the Chinese Academy of Sciences. K562, C-4I, SW756, MS751, U2OS, LOVO, HGC27, A549 and PANC-1 cell lines were purchased from ATCC. HLA-DRB1*09:01 or HPV18E7 transduced cell line were established in house with lentivirus transduction and selected with puromycin or G418. EBV-LCLs were established by immortalization of B cells from PBMCs of patients and healthy donors using supernatant from the cell line B95.8.

Authentication

All the cell line used in this study have been validated by STR.

Mycoplasma contamination

All cell lines are free of Mycoplasma contamination. Cells were regularly tested for mycoplasma.

Commonly misidentified lines
(See ICLAC register)

No commonly misidentified cell lines were used in the study

Animals and other research organisms

Policy information about studies involving animals; ARRIVE guidelines recommended for reporting animal research, and Sex and Gender in Research

Laboratory animals

NOG (NOD.Cg-PrkdcscidIL2rgtm1Sug/JicCrl) mice (female, 6-8 weeks old) were purchased from Beijing Vital River Laboratory Animal Technology Co., Ltd. All the experimental mice were housed under controlled temperature (21–23 °C) and 12:12 light: dark cycle conditions with free access to standard diet and water.

Wild animals

No wild animals and field collected samples were used in the study.

Reporting on sex

Both male and female mice were used in the study.

Field-collected samples	*For laboratory work with field-collected samples, describe all relevant parameters such as housing, maintenance, temperature, photoperiod and end-of-experiment protocol OR state that the study did not involve samples collected from the field.*
Ethics oversight	Animal research protocols were approved by the Institutional Animal Care and Use Committee (IACUC) of Guangzhou Curegenix Inc (IACUC#YSDW202203016-2)

Note that full information on the approval of the study protocol must also be provided in the manuscript.

Plants

Seed stocks	*Report on the source of all seed stocks or other plant material used. If applicable, state the seed stock centre and catalogue number. If plant specimens were collected from the field, describe the collection location, date and sampling procedures.*
Novel plant genotypes	*Describe the methods by which all novel plant genotypes were produced. This includes those generated by transgenic approaches, gene editing, chemical/radiation-based mutagenesis and hybridization. For transgenic lines, describe the transformation method, the number of independent lines analyzed and the generation upon which experiments were performed. For gene-edited lines, describe the editor used, the endogenous sequence targeted for editing, the targeting guide RNA sequence (if applicable) and how the editor was applied.*
Authentication	*Describe any authentication procedures for each seed stock used or novel genotype generated. Describe any experiments used to assess the effect of a mutation and, where applicable, how potential secondary effects (e.g. second site T-DNA insertions, mosiacism, off-target gene editing) were examined.*

Flow Cytometry

Plots

Confirm that:

- [ ] The axis labels state the marker and fluorochrome used (e.g. CD4-FITC).
- [x] The axis scales are clearly visible. Include numbers along axes only for bottom left plot of group (a 'group' is an analysis of identical markers).
- [ ] All plots are contour plots with outliers or pseudocolor plots.
- [ ] A numerical value for number of cells or percentage (with statistics) is provided.

Methodology

Sample preparation	For surface staining, 1 × 105~1 × 106 t cells were collected and washed with FACS buffer (PBS containing 2% FBS). Surface staining antibodies were added and incubated at 4°C for 30 minutes. After washing, samples were resuspended in FACS buffer for analysis by flow cytometer.<br><br>For intracellular cytokine staining, t cells were co-cultured with target cells at E:T ratio 1:1 for 4 hours in the presence of GolgiPlug (BD Biosciences, cat. 00-4506-51). After co-cultured, surface markers were added to label cells for 30 minutes at 4° C. These T cells were fixed, permeabilized in permeabilizing buffer (BD Biosciences, 554714) and stained with intracellular markers. Samples were acquired on BD FACS Canto II flow cytometer or Thermo Attune® NxT flow cytometer, and data were analyzed by using the Flowjo software.
Instrument	BD FACS Canto II flow cytometer or Attune NxT flowcytometer
Software	FACS data were analyzed with Flowjo software v10.8.1
Cell population abundance	As described in the method section
Gating strategy	As described in the method section

- [ ] Tick this box to confirm that a figure exemplifying the gating strategy is provided in the Supplementary Information.
